# Structural and Visual Changes in Branch Retinal Vein Occlusion Patients with Retinal Atrophy

**DOI:** 10.1155/2022/8945467

**Published:** 2022-08-18

**Authors:** Zhenping Li, Xiaoya Gu, Shuang Song, Xiaobing Yu, Peng Zhang, Hong Dai

**Affiliations:** Department of Ophthalmology, Beijing Hospital, National Centre of Gerontology, Institute of Geriatric Medicine, Chinese Academy of Medical Sciences, Beijing, China

## Abstract

**Background:**

To study the changes of retinal vascular density (VD), retinal thickness (RT), and their correlations with visual acuity (VA) in branch retinal vein occlusion (BRVO) patients with retinal atrophy after resolution of macular oedema (MO).

**Methods:**

This is a retrospective study consisting of 46 patients diagnosed with BRVO at Beijing Hospital from 2015 to 2019. Patients' 46 affected eyes and 39 fellow eyes were included. The affected eyes were further divided into the atrophy group and the nonatrophy group. Optical coherence tomography (OCT) and optical coherence tomography angiography (OCTA) images obtained when MO completely resolved after treatment with ranibizumab were analyzed. We quantitatively measured foveal avascular zone (FAZ) parameters, the disruption extent of ellipsoid zone (EZ), RT, and VD of superficial vascular complex (SVC), and deep vascular complex (DVC) in central fovea and the minimal-VD quadrant. Paired *t*-tests, independent *t*-tests, Mann–Whitney *U* tests, Wilcoxon tests, Pearson correlation analyses, Spearman correlation analyses, and single and multiple linear regression models were adopted.

**Results:**

Compared with nonatrophy eyes, the minimal-VD quadrantal (quadrantal for short) SVC-VD (25.76 ± 4.57% versus 36.21 ± 6.47%, *P* < 0.001) and quadrantal DVC-VD (27.72 (17.23) % versus 38.95 (11.05) %, *P* = 0.001) of atrophy eyes decreased significantly. Quadrantal SVC-VD and quadrantal DVC-VD were strongly correlated with quadrantal full RT (*r* = 0.763 and 0.698, both *P* < 0.001). The disruption length of EZ was significantly correlated with quadrantal full RT (*r* = −0.406, *P* = 0.005) and quadrantal SVC-VD (*r* = −0.298, *P* = 0.044). In multiple linear regression analysis, the disruption length of EZ and VA before treatment and age showed significant correlations with VA with complete resolution of MO (*P* = 0.020,  0.033,  and 0.002).

**Conclusions:**

The retinal VD on the affected area correlates well with the corresponding full RT when BRVO-MO completely resolves, suggesting that VD may predict the final RT. Severe decrease in VD may result in retinal atrophy, which may cause VA loss indirectly with the intermediate influencing factor of EZ defects.

## 1. Background

Branch retinal vein occlusion (BRVO) is a subgroup of retinal vein occlusion (RVO) with a prevalence rate of 0.442% in people over 30 years old in the world [1, 2]. Macular oedema (MO) secondary to branch retinal vein occlusion usually causes visual impairment. Fundus fluorescein angiography (FFA) is considered the gold standard for RVO diagnosis [2]. Compared with FFA, optical coherence tomography angiography (OCTA) has the advantages of segregating the deep and superficial retinal capillary network and quantifying their vascular density (VD) using noninvasive methods [3–5]. Intravitreal injection of antivascular endothelial growth factor (VEGF) drugs is commonly used to treat BRVO-MO. However, MO often recurs and needs frequent injections. At present, factors related to BRVO-MO recurrence are still unclear [6–8].

Retinal thickness (RT) often increases because of MO and returns to normal level after anti-VEGF treatment [9, 10]. However, it was sometimes observed that RT may decrease to a lower level than the normal control eye after MO resolution. Using FFA, optical coherence tomography (OCT), and histopathology, some researchers found that severe ischemia might be the cause of retinal atrophy [11, 12]. However, they failed to establish a linear correlation between retinal VD and RT. It was shown in many researches that BRVO can reduce retinal VD [13, 14]. Deng et al. [15] and Kim et al. [16] used OCTA to study the correlations between visual acuity (VA), central retinal thickness (CRT), and VD of superficial complex plexus (SCP) and deep complex plexus (DCP) in BRVO-MO patients after one anti-VEGF injection. MO may not completely resolve after one anti-VEGF injection; hence, the effects of oedema on the segregation of different retinal layers and VD quantitative measurement cannot be ruled out. In addition, RT without any MO can truly represent the functional changes after the onset of BRVO. Therefore, it is important for us to study the correlations between VA, RT, and retinal VD after complete resolution of MO.

Some studies suggested that retinal atrophy caused by severe ischemia resulted in a lower recurrence rate or less severity of MO, indicating that atrophy might lead to anatomic and functional changes of retina [17–19]. Podkowinski defined retinal atrophy as RT < 260 *μ*m outside the foveal center [20]. In this study, we included BRVO-MO patients whose MO completely resolved after several intravitreal ranibizumab (IVR) injections and quantitatively analyzed retinal VD, RT, and their correlations. We then further analyzed their relationships with VA and oedema recurrence. Factors affecting VA were also explored.

## 2. Methods

### 2.1. Participants

Forty-six patients with BRVO enrolled in Song's study were included. Song's study was a 12-month randomized controlled study assessing the efficacy of intravitreal ranibizumab in patients with ME secondary to BRVO [21]. This trial conformed to the principles of the Declaration of Helsinki. Inclusion criteria were as follows: (1) the affected eye was diagnosed with MO secondary to BRVO by slit-lamp biomicroscopy, OCT, OCTA, and FFA at the first visit from 2015 to 2019; (2) CRT measured by OCT before treatment was ≥300 *μ*m; (3) complete resolution of MO can be observed during 12-months follow-up. Exclusion criteria were as follows: (1) eyes with refractive media opacity affecting fundus imaging such as vitreous hemorrhage or severe cataract; (2) eyes with macular epiretinal membrane or fundus vascular diseases; (3) eyes with OCTA image quality < 7. Forty-six affected eyes were included, and thirty-nine fellow eyes were used as control. The remaining 7 fellow eyes lacked available images. All patients were followed up for 12 months and underwent OCTA and OCT examinations at each visit. During the follow-up, 3 initial IVR (Lucentis 0.5 mg) injections must be conducted monthly in all affected eyes, and additional injections would be repeated if VA suffered more than 5 letters loss due to disease activity or CRT increased more than 100 *μ*m compared to the previous visit. All patients had blood pressure (BP) measurement at each visit, and the BP was normal in hypertension patients during the follow-up time [21].

### 2.2. Grouping

Retinal atrophy was defined as the minimal-VD quadrantal (quadrantal for short) full RT < 260 *μ*m, which was similar to Podkowinski [20]. This was based on reported normal RT with Iowa Reference Algorithm [22]. We divided the affected eyes into the nonatrophy group (quadrantal full RT ≥ 260 *μ*m, *n* = 32) and the atrophy group (quadrantal full RT < 260 *μ*m, *n* = 14). Thirty-nine fellow eyes were defined as the control group.

### 2.3. Optical Coherence Tomography Angiography (OCTA)

Spectral-domain OCTA (RTVue XR Avanti, Optovue, Inc., Fremont, CA, USA) was used. We collected images with complete resolution of MO near the end of follow-up, and the complete resolution of MO was defined as 304 × 304 B-scans within a 3 mm × 3 mm area centered on fovea on OCTA did not show any cysts or retinal thickening. Retinal vasculature was automatically divided into superficial vascular complex (SVC) and deep vascular complex (DVC). SVC included vasculature from internal limiting membrane (ILM) to −10 *μ*m below the inner plexiform layer (IPL). DVC included vasculature from −10 *μ*m below the IPL to 10 *μ*m below the outer plexiform layer (OPL). Full RT included retinal layers from ILM to retinal pigmented epithelial (RPE), and superficial RT included retinal layers from ILM to −10 *μ*m below the IPL. We subtracted these two to obtain deep RT. According to the Early Treatment Diabetic Retinopathy Study (ETDRS) ring, 3 mm × 3 mm images centered on fovea were divided into central foveal zone (1 mm diameter) and 4 parafoveal quadrants between the 1 mm diameter and 3 mm diameter rings (temporal, superior, nasal, and inferior). All data of RT and VD were processed by the AngioVue software (version 2017.1.0.151, Optovue, Inc.). We selected the minimal-VD quadrant and central foveal zone. The corresponding areas of fellow eyes were also included in this study. We collected the data on foveal avascular zone (FAZ) area, FAZ perimeter, FAZ avascular index (AI: FAZ perimeter/perimeter of equal area standard circumference), and FD-300-VD (VD within a 300 *μ*m wide ring around the FAZ).

### 2.4. Optical Coherence Tomography (OCT)

Spectral-domain OCT (SPECTRALIS HRA + OCT, Heidelberg Engineering, Heidelberg, Germany) was used. OCT images on the same occasion as OCTA images were included. Among 6 B-scans passing through the foveal center, two experienced physicians masked to the study process manually measured the longest disruption length of the ellipsoid zone within 1 mm diameter range of the central fovea, and the average was taken for study.

### 2.5. Visual Acuity

Best corrected visual acuity (BCVA) was measured with an ETDRS chart. Patient's BCVA was measured before treatment and at every follow-up visit.

### 2.6. Statistical Analysis

Statistical analyses were performed using SPSS 25.0 software, and statistical significance was established at two-tailed *P* < 0.05. Continuous variables with normal distribution were presented as mean ± standard deviation and tested with paired *t*-tests (affected eyes versus fellow eyes) or independent *t*-tests (nonatrophy versus atrophy), continuous variables with nonnormal distribution were presented as median (interquartile range) and tested with Wilcoxon paired signed rank test (affected eyes versus fellow eyes) or Mann–Whitney *U* tests (nonatrophy versus atrophy), and ranked variables were tested with Chi-squared tests. Normality of errors (residuals) was checked while modeling (histograms and P–P plots). Pearson correlation analyses were adopted to study the correlations among VD, RT, FAZ area, EZ disruption length, and visual acuity (errors were normally distributed). Spearman correlation analyses were adopted to study the correlations between the number of IVR injections and VD and RT (errors were not normally distributed). Single and multiple linear regression models were calculated using BCVA with complete resolution of MO as a dependent variable and potential relative parameters as predictors.

## 3. Results

We studied 46 affected eyes and 39 fellow eyes of 46 patients, including 20 men and 26 women. The mean age of the patients was 59.07 ± 10.80 years. All the affected eyes were diagnosed as MO secondary to BRVO at the first visit, and MO resolved completely during follow-up.

Table 1 shows comparisons between affected eyes and fellow eyes. The quadrantal full RT of affected and fellow eyes were 283.32 ± 35.68 *μ*m and 319.20 ± 15.12 *μ*m, respectively, and the difference was significant (*P* < 0.001). Compared with the fellow eyes, the quadrantal superficial RT, quadrantal deep RT, foveal full RT, quadrantal SVC-VD, quadrantal DVC-VD, foveal SVC-VD, foveal DVC-VD, and FD-300-VD of affected eyes decreased significantly (all *P* < 0.05). The FAZ area, perimeter, and AI of affected eyes increased significantly (all *P* < 0.05).

Table 2 shows comparisons between atrophy eyes and nonatrophy eyes. The demographic characteristics, incidence of hypertension and diabetes, disease duration, and number of IVR injections before complete resolution of MO were not significantly different between these two groups. Compared with nonatrophy eyes, the quadrantal superficial RT, quadrantal deep RT, and foveal full RT of atrophy eyes decreased significantly (all *P* < 0.05). The quadrantal SVC-VD (25.76 ± 4.57% versus 36.21 ± 6.47%, *P* < 0.001), quadrantal DVC-VD (27.72 (17.23) % versus 38.95 (11.05) %, *P*=0.001), and FD-300-VD (40.10 (6.53) % versus 46.22 (7.10) %, *P* < 0.001) of atrophy eyes decreased significantly compared with nonatrophy eyes. The disruption length of EZ of the atrophy group was longer than that of the nonatrophy group (605.50 (678) um versus 355.50 (567) um), but the difference was not significant (*P*=0.218). The FAZ area, FAZ perimeter, and FAZ AI were not significantly different between these two groups (all *P* > 0.05). In the atrophy group, VA with complete resolution of MO (72.50 (23.00) versus 79.00 (11.00)) and improved VA (15.93 ± 8.41 versus 17.84 ± 8.57) were lower than those of the nonatrophy group, but the differences were not significant (all *P* > 0.05). The number of IVR injections during 12 months was not significantly different between the two groups (*P* > 0.05).

**Figure 1:**
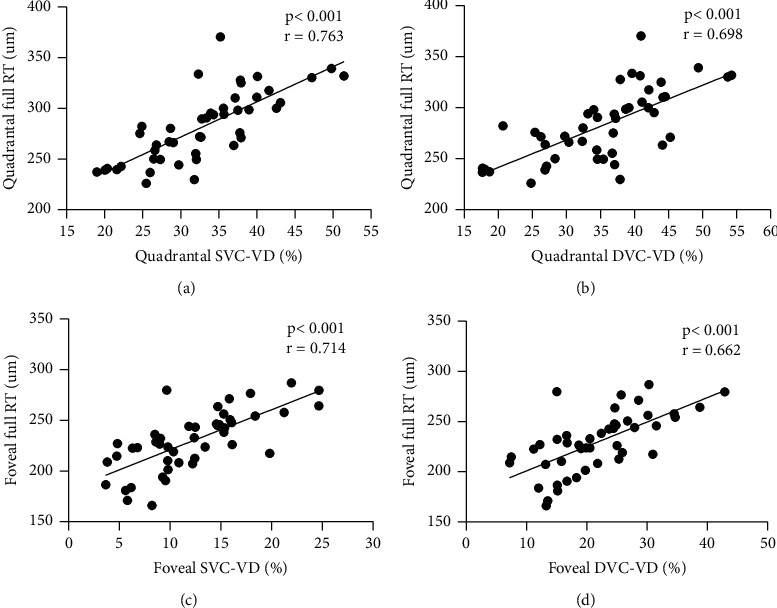
Scatter plots show correlations between VD and RT in the minimal-VD quadrant and fovea. SVC: superficial vascular complex; DVC: deep vascular complex; VD: vascular density; RT: retinal thickness; the minimal-VD quadrantal: quadrantal for short.

**Figure 2:**
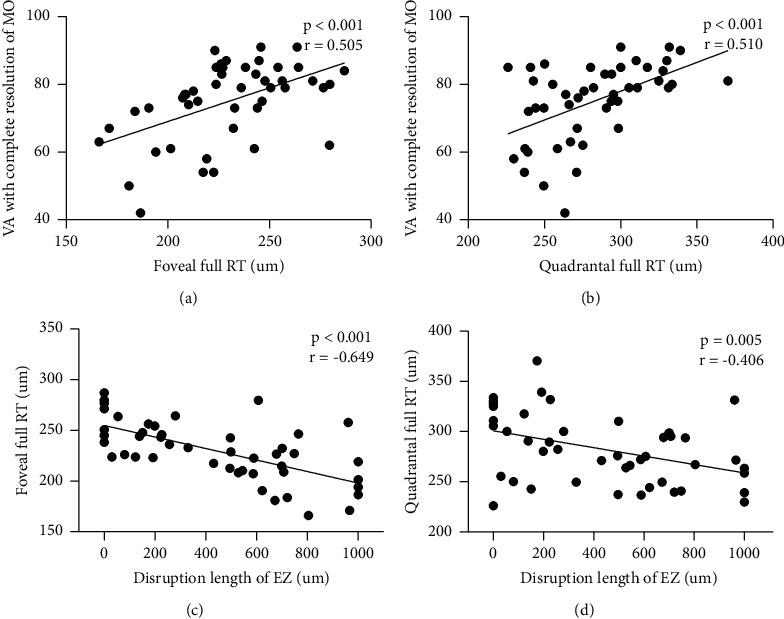
Correlations between VA with complete resolution of MO and disruption length of EZ and RT. VA: visual acuity; MO: macular oedema; RT: retinal thickness; EZ: ellipsoid zone; the minimal-VD quadrantal: quadrantal for short.

Tables 3 and 4 show Pearson correlations between different variables. Quadrantal SVC-VD and quadrantal DVC-VD were strongly correlated with quadrantal full RT (*r* = 0.763 and 0.698, both *P* < 0.001). Foveal SVC-VD and foveal DVC-VD were strongly correlated with foveal full RT (*r* = 0.714 and 0.662, both *P* < 0.001) (Figure 1). The disruption length of EZ was significantly correlated with foveal full RT (*r* = −0.649, *P* < 0.001), quadrantal full RT (*r* = −0.406, *P*=0.005) (Figure 2), foveal SVC-VD (*r* = −0.454, *P*=0.002), foveal DVC-VD (*r* = −0.379, *P*=0.009), and quadrantal SVC-VD (*r* = −0.298, *P*=0.044). VA with complete resolution of MO was significantly correlated with foveal full RT (*r* = 0.505, *P* < 0.001), quadrantal full RT (*r* = 0.510, *P* < 0.001) (Figure 2), and quadrantal SVC-VD (*r* = 0.347, *P*=0.018). The number of IVR injections during 12 months showed no significant correlations with RT and VD in quadrant and fovea.

In simple linear regression analysis, quadrantal SVC-VD, quadrantal full RT, foveal full RT, and the disruption length of EZ and VA before treatment were found to have significant correlations with VA with complete resolution of MO (all *P* < 0.05) (Appendix 1). After exclusion of collinearity, quadrantal SVC-VD, quadrantal full RT, and the disruption length of EZ and VA before treatment were included in multiple linear regression analysis model as predictors. Additionally, age [23, 24] and FAZ area [25–27] were reported to have significant correlations with VA; thus, they were also included. In multiple linear regression analysis, age and the disruption length of EZ and VA before treatment showed significant correlations with VA with complete resolution of MO (*P*=0.020,  0.033,  and 0.002), while FAZ area, quadrantal SVC-VD, and quadrantal full RT did not (all *P* > 0.05) (Table 5).

## 4. Discussion

Little is known about the structural changes using OCTA in eyes with BRVO-MO that achieved resolution of macular oedema. Podkowinski defined retinal atrophy as RT < 260 *μ*m outside the foveal center [20]. This was based on reported normal RT with Iowa Reference Algorithm [22]. In the present study, retinal atrophy was defined as the minimal-VD quadrantal (quadrantal for short) full RT < 260 *μ*m, which was similar to Podkowinski.

In this study, we found that quadrantal SVC-VD and quadrantal DVC-VD in the atrophic group were significantly lower than those in the nonatrophic group and that all the quadrantal and foveal SVC-VD and DVC-VD were strongly positively correlated with the corresponding full RT. We suggest that a decrease in VD may lead to a corresponding decrease in RT in BRVO eyes with complete resolution of MO. The more severe the retinal ischemia is, the thinner the corresponding retina is. Our conclusions are similar to others; Yeung found that inner retinal loss (ILM to the outside border of INL) in DR patients was highly correlated with a capillary nonperfused area on FFA [11]. Pathological study suggested that arterial plexus destruction caused by RVO would result in hypoxia-induced cell death, loss of IPL and INL, and finally retinal atrophy [12]. These two reports have used FFA, OCT, and histopathological way to infer that severe ischemia may lead to inner retinal atrophy. Hasegawa speculated that a large reduction in VD on OCTA might lead to inner retinal atrophy in BRVO [28]. The strength of our study is using OCTA to quantitatively measure VD and confirming a strong, linear, and positive correlation between VD and RT. It was known that the inner retina was supplied by the retinal blood system, and the outer retina was supplied by the choroidal blood system. We confer that obstruction in branch retinal vein will damage capillaries network and release more VEGF, resulting in cellular apoptotic and necrotic changes due to lack of oxygen and nutrition. The increase of VEGF and breakage of the blood-retinal barrier (BRB) will cause the accumulation of intraretinal cystic oedema. After injections of anti-VEGF drugs, the cystic oedema resolves, dead cells are phagocytosed, and retina becomes thin and even atrophic. But sometimes, retinal atrophy may be hidden behind the macular oedema, and its formation can be a gradual process [20]. Thin or atrophic retina will never return to normal thickness owing to the low possibility of recanalization of retinal vessels and irreversible damage of cells. Thus, VD at the onset of BRVO-MO helps to predict the final RT outcome when MO resolves later.

FAZ area, FAZ perimeter, FAZ AI, FD-300-VD, foveal SVC-VD, and foveal DVC-VD were often considered indexes of macular ischemia [29]. We found that only FD-300-VD in atrophy eyes significantly decreased compared with nonatrophy eyes. FAZ indexes, like FAZ area, FAZ perimeter, and FAZ AI, represent ischemia of the whole central macular area, which were also affected by other nonischemic quadrants in BRVO patients. This may explain why retinal atrophy was not correlated to FAZ indexes in BRVO patients. We found that retinal thinning happened simultaneously in superficial and deep layers of retina, which was similar to the previous reports. Podkowinski found that all 3 different retinal compartment layers were thinner in the atrophy group than in the control group [20]. Hasegawa found that low reflective spaces in the retinal nerve fibre layer could predict full RT and inner RT thinning in BRVO patients [19]. Our findings indicate that necrotic or apoptotic changes of cells simultaneously happened in superficial and deep retinal layers due to a decrease in both SVC-VD and DVC-VD. Thus, we should not focus on a single layer of BRVO eyes.

We found that the total number of IVR injections within 12 months showed no significant difference between the atrophy group and the nonatrophy group. Additionally, it had no significant correlations with RT and VD, suggesting that recurrences and severity of MO were not significantly related to retinal vasculature loss and retinal thinning. However, several previous reports showed that severe ischemia and retinal atrophy might lead to fewer injections of anti-VEGF drugs and less severity of MO. Hasegawa found that the reduction in macular VD was associated with a decrease in the number of MO recurrences [28]; Finkelstein suggested that ischemic MO was often transient and resolved spontaneously, while well-perfused MO persisted [17]; Sakimoto found that MO occurred more frequently in partially perfused areas rather than nonperfused areas [18]. It is speculated that wide nonperfused areas may lead to thinning and atrophy of the inner retina, reduce oxygen consumption, achieve a balance of supply and demand for oxygen, and finally reduce the secretion of VEGF and fluid leakage [18, 28, 30, 31]. The difference might be due to the fact that we treated BRVO patients with 3 initial monthly ranibizumab injections and criteria-driven pro re nata (PRN) injections, while Hasegawa treated patients with one initial injection and retreated if the central foveal thickness was ≥300 *μ*m. And we only focused on the 3 mm × 3 mm macular area on OCTA, while Finkelstein and Sakimoto focused on a wider area far away from the fovea on FFA.

In the single linear regression model, we found that VA with complete resolution of MO was positively correlated with quadrantal SVC-VD, but not quadrantal DVC-VD. The reason might be apoptotic or necrotic changes of RNFL, which receives its blood supply from SVC and plays an important role in VA [19]. And the reason why we could not find a significant correlation between VA and DVC-VD might be the small sample in our study and the fluctuation of the nature of OCTA readings. Winegarner [32] and Deng [15] came to a similar conclusion that VA correlated positively significantly with both parafoveal SCP-VD and DCP-VD after anti-VEGF treatment for RVO-MO. Kim found a significant positive correlation between VA and parafoveal DCP-VD after one anti-VEGF treatment for BRVO [16]. However, Kim and Deng only performed a single linear regression analysis and did not perform a multiple linear regression analysis further. In Winegarner's study, posttreatment DCP-VD was significantly associated with posttreatment VA in multiple linear regression analysis, but this model did not include EZ disruption. It is difficult to tell whether the factor truly affecting visual acuity is the change of VD or EZ disruption. Additionally, significant positive correlations between VA with complete resolution of MO and both quadrantal full RT and foveal full RT were discovered, suggesting that the thinner the quadrantal full-thickness RT and foveal full RT are, the worse the VA with complete resolution of MO may be. Deng concluded, in contrast to us, that they found significant negative correlations between VA and CRT and parafoveal RT in BRVO patients after one anti-VEGF treatment, possibly because some BRVO eyes remained edematous [15].

In the multiple linear regression model, we found no significant correlations between quadrantal SVC-VD and quadrantal full RT and VA with complete resolution of MO, suggesting that they might not be directly correlated with VA. Additionally, we found that VA before treatment, the disruption length of EZ, and age were all significantly correlated with VA with complete resolution of MO. These were similar to Nakano [23]. Some studies have reported that the destruction of EZ would lead to decreased visual acuity [23, 33]. Photoreceptors in the central foveal are mainly cone photoreceptors, which play an essential role in photopic vision [34]. EZ is considered photoreceptor inner segments in anatomic location [35], and its integrity has been reported as a key factor affecting visual acuity in many retinal conditions, including BRVO [35]. EZ is mainly supplied by choroidal vessels, while the changes of VD, RT, and FAZ indexes reflect the changes of retinal vessels. And its disruption is usually caused by macular oedema in addition to poor retinal perfusion in BRVO. A possible mechanism is that reduction of retinal VD leads to retinal thinning or atrophy, which is likely to be simultaneously accompanied by the defects of the EZ in the BRVO pathological process. We speculate that the relationships between retinal VD, RT, and EZ disruption and VA loss are concomitant. The quadrantal full RT and quadrantal SVC-VD showed significant correlations with the disruption length of EZ, which might be the intermediate influencing factor between VD, RT, and VA. This may account for the plausible effects of VD and RT on VA in single linear regression models. Among retinal VD, RT, and EZ disruption, only EZ disruption might be the key factor affecting VA. In addition, it has been reported that loss of foveal bulge of EZ in BRVO led to lower BCVA [36]. Therefore, in addition to focusing on changes in retinal full RT and VD with complete resolution of MO, we have to concentrate more on the structural changes of EZ.

The limitations of our study are as follows. Firstly, this is a retrospective study with possible selection bias and a rather small sample size. Further prospective study should be conducted to verify the conclusion. Secondly, we only analyzed the image data when MO was completely resolved, lacking dynamic observation during the disease course. Thirdly, the software we used is an upgraded version, which is smarter than the previous version and can reduce the presence of vascular projection artifacts. We segmented retinal layers when the macular oedema completely resolved. But it is not excluded that there are still a small number of projection artifacts and layer segmentation errors. Finally, we only measured retinal VD and RT in 3 mm × 3 mm area in fovea, which did not reflect the whole data of retina.

In conclusion, the retinal VD on the affected area correlates well with the corresponding full RT when BRVO-MO completely resolves after anti-VEGF treatment, suggesting that VD at the onset of the disease course may predict the final RT outcome. Severe decrease in VD may even result in retinal atrophy, which may cause VA loss indirectly with the intermediate influencing factor of EZ defects. The VA before treatment, the disruption extent of EZ, and age may be significant factors affecting the VA with complete resolution of MO.

## Figures and Tables

**Table 1:** Characteristics of affected versus fellow eyes.

Variables	Affected eyes (*n* = 39)	Fellow eyes (*n* = 39)	*P* value
FAZ area (mm^2^)	0.37 (0.25)	0.33 (0.15)	0.003
FAZ perimeter (mm)	2.50 (0.85)	2.31 (0.56)	0.002
FAZ AI	1.17 (0.12)	1.13 (0.06)	0.003
FD-300-VD (%)	43.36 ± 7.47	50.05 ± 2.98	<0.001
Quadrantal SVC-VD (%)	33.88 ± 7.79	49.62 ± 3.26	<0.001
Quadrantal DVC-VD (%)	36.33 ± 8.78	51.66 ± 3.54	<0.001
Foveal SVC-VD (%)	12.78 ± 5.49	16.88 ± 6.07	<0.001
Foveal DVC-VD (%)	22.53 ± 8.27	29.05 ± 6.48	<0.001
Quadrantal superficial RT (*μ*m)	87.68 ± 18.90	108.05 ± 11.47	<0.001
Quadrantal deep RT (*μ*m)	195.64 ± 24.41	211.16 ± 8.91	<0.001
Quadrantal full RT (*μ*m)	283.32 ± 35.68	319.20 ± 15.12	<0.001
Foveal full RT (*μ*m)	229.99 ± 29.18	244.68 ± 21.40	<0.001
VA before treatment	57.26 ± 10.27	82.10 ± 8.01	<0.001
VA with complete resolution of MO	74.72 ± 12.40	82.10 ± 8.01	0.001

All values are presented as mean ± SD or median (IQR). FAZ: foveal avascular zone; AI: acircularity index; FD-300-VD: vascular density within a 300 *μ*m wide ring around the foveal avascular zone; SVC: superficial vascular complex; DVC: deep vascular complex; VD: vascular density; RT: retinal thickness; VA: visual acuity; MO: macular oedema; the minimal-VD quadrantal: quadrantal for short; SD: standard deviation; IQR: interquartile range.

**Table 2:** Characteristics of non-atrophy versus atrophy eyes.

Variables	Nonatrophy (*n* = 32)	Atrophy (*n* = 14)	*P* Value
Age (years)	59.41 ± 10.71	58.29 ± 11.38	0.750
Sex (male/female)	19/13	9/5	0.754
Eye (OD/OS)	15/17	5/9	0.482
Hypertension (±)	15/17	5/9	0.482
Diabetes (±)	5/27	1/13	0.756
Duration from onset to 1st IVR (months)	1.00 (2.00)	1.00 (2.00)	0.960
Duration from 1st IVR to complete resolution of MO (months)	10.00 (5.00)	11.00 (3.00)	0.196
Duration from onset to complete resolution of MO (months)	11.00 (3.00)	12.00 (3.00)	0.317
Number of IVR injections before complete resolution of MO	3.00 (2.00)	4.00 (3.00)	0.610
FAZ area (mm^2^)	0.37 (0.26)	0.38 (0.23)	0.747
FAZ perimeter (mm)	2.51 (0.91)	2.49 (0.70)	0.747
FAZ AI	1.17 (0.15)	1.17 (0.09)	0.738
FD-300-VD (%)	46.22 (7.10)	40.10 (6.53)	<0.001
Quadrantal SVC-VD (%)	36.21 ± 6.47	25.76 ± 4.57	<0.001
Quadrantal DVC-VD (%)	38.95 (11.05)	27.72 (17.23)	0.001
Foveal SVC-VD (%)	12.87 ± 5.72	10.76 ± 4.07	0.219
Foveal DVC-VD (%)	23.09 ± 8.81	19.09 ± 5.03	0.120
Quadrantal superficial RT (*μ*m)	92.69 ± 17.55	69.92 ± 7.13	<0.001
Quadrantal deep RT (*μ*m)	206.77 ± 20.43	172.87 ± 11.49	<0.001
Foveal full RT (*μ*m)	235.58 ± 30.60	216.46 ± 22.25	0.041
Disruption length of EZ	355.50 (567)	605.50 (678)	0.218
VA before treatment	61.00 (10.00)	53.50 (19.00)	0.092
VA with complete resolution of MO	79.00 (11.00)	72.50 (23.00)	0.062
Improved VA	17.84 ± 8.57	15.93 ± 8.41	0.487
Number of IVR injections during 12 months	4.50 (3.00)	4.00 (3.00)	0.958

All values are presented as mean ± SD or median (IQR). MO: macular oedema; FAZ: foveal avascular zone; AI: acircularity index; FD-300-VD: vascular density within a 300 *μ*m wide ring around the foveal avascular zone; SVC: superficial vascular complex; DVC: deep vascular complex; VD: vascular density; RT: retinal thickness; EZ: ellipsoid zone; VA: visual acuity; the minimal-VD quadrantal: quadrantal for short; SD: standard deviation; IQR: interquartile range.

**Table 3:** Correlations between vascular density and retinal thickness with complete resolution of macular oedema.

Characteristics	Correlation	Quadrantal SVC-VD	Quadrantal DVC-VD	Foveal SVC-VD	Foveal DVC-VD
Quadrantal full RT	N	46	46	46	46
	*P* value	<0.001	<0.001	0.088	0.033
	r coefficient	0.763	0.698	0.254	0.314

Foveal full RT	N	46	46	46	46
	*P* value	0.075	0.038	<0.001	<0.001
	r coefficient	0.265	0.307	0.714	0.662

SVC: superficial vascular complex; DVC: deep vascular complex; VD: vascular density; RT: retinal thickness; the minimal-VD quadrantal: quadrantal for short.

**Table 4:** Correlations between VD, RT, and EZ disruption length, FAZ area, and VA and IVR injections number with complete resolution of macular oedema.

Characteristics	Correlation	Quadrantal SVC-VD	Quadrantal DVC-VD	Quadrantal full RT	Foveal SVC-VD	Foveal DVC-VD	Foveal full RT
Disruption length of EZ	N	46	46	46	46	46	46
	*P* value	0.044	0.064	0.005	0.002	0.009	<0.001
	r coefficient	−0.298	−0.276	−0.406	−0.454	−0.379	−0.649

FAZ area	N	46	46	46	46	46	46
	*P* value	0.341	0.228	0.532	<0.001	<0.001	0.004
	r coefficient	−0.143	−0.181	−0.095	−0.520	−0.578	−0.417

VA with complete resolution of MO	N	46	46	46	46	46	46
	*P* value	0.018	0.142	<0.001	0.061	0.123	<0.001
	r coefficient	0.347	0.220	0.510	0.279	0.231	0.505

Number of IVR injections during 12 months (^*∗*^)	N	46	46	46	46	46	46
	*P* value	0.952	0.366	0.450	0.509	0.286	0.987
	r coefficient	−0.009	−0.138	−0.116	0.101	0.162	0.003

(^*∗*^): spearman correlation analysis. FAZ: foveal avascular zone; SVC: superficial vascular complex; DVC: deep vascular complex; VD: vascular density; RT: retinal thickness; EZ: ellipsoid zone; VA: visual acuity; MO: macular oedema; the minimal-VD quadrantal: quadrantal for short.

**Table 5:** Multiple linear regression models for VA with complete resolution of macular oedema.

	95% CI		Estimated	*t* value	*P* value
	Lower	Upper			
Age	−0.465	−0.042	−0.236	−2.428	0.020
FAZ area	−10.646	7.496	−0.035	−0.351	0.727
Quadrantal SVC-VD	−0.551	0.387	−0.054	−0.354	0.726
Quadrantal full RT	−0.040	0.184	0.216	1.305	0.199
Disruption length of EZ	−0.019	−0.001	−0.280	−2.212	0.033
VA before treatment	0.202	0.830	0.436	3.321	0.002

FAZ: foveal avascular zone; SVC: superficial vascular complex; VD: vascular density; RT: retinal thickness; EZ: ellipsoid zone; VA: visual acuity; the minimal-VD quadrantal: quadrantal for short.

## Data Availability

The datasets of the study are available from the corresponding author upon reasonable request.

## Supplementary Materials

Supplementary Materials Additional file 1: Appendix 1. Single linear regression models for VA with complete resolution of macular oedema.
