# Supplementary material for: Structural and Visual Changes in Branch Retinal Vein Occlusion Patients with Retinal Atrophy
Source: J Ophthalmol. 2022 Aug 18;2022:8945467. doi: 10.1155/2022/8945467 (PMC9410836; doi:10.1155/2022/8945467)
Supplement: Supplementary Materials — Additional file 1: Appendix 1. Single linear regression models for VA with complete resolution of macular oedema. [file 8945467.f1.docx]

| **Appendix 1.** Single linear regression models for VA with complete resolution of macular oedema | | | | |
| --- | --- | --- | --- | --- |
|  | Estimated | SE | R^2^ | P value |
| Age | -0.263 | 0.157 | 0.060 | 0.101 |
| FAZ area | -8.500 | 6.674 | 0.036 | 0.210 |
| FAZ perimeter | -1.838 | 1.775 | 0.024 | 0.306 |
| FAZ AI | -3.836 | 17.131 | 0.001 | 0.824 |
| Quadrantal SVC-VD | 0.526 | 0.215 | 0.120 | 0.018 |
| Quadrantal DVC-VD | 0.283 | 0.189 | 0.048 | 0.142 |
| Foveal SVC-VD | 0.608 | 0.316 | 0.078 | 0.061 |
| Foveal DVC-VD | 0.334 | 0.212 | 0.053 | 0.123 |
| Quadrantal full RT | 0.170 | 0.043 | 0.260 | ＜0.001 |
| Foveal full RT | 0.199 | 0.051 | 0.255 | ＜0.001 |
| Disruption length of EZ | -0.022 | 0.004 | 0.411 | ＜0.001 |
| VA before treatment | 0.826 | 0.127 | 0.489 | ＜0.001 |
| FAZ: foveal avascular zone; AI: acircularity index; SVC: superficial vascular complex; DVC: deep vascular complex; VD: vascular density; RT: retinal thickness; EZ: ellipsoid zone; VA: visual acuity; the minimal-VD quadrantal: quadrantal for short. | | | | |
|  |  |  |  |  |
|  |  |  |  |  |
